# The PARN, TOE1, and USB1 RNA deadenylases and their roles in non-coding RNA regulation

**DOI:** 10.1016/j.jbc.2023.105139

**Published:** 2023-08-06

**Authors:** Thao Ngoc Huynh, Roy Parker

**Affiliations:** 1 Department of Biochemistry, University of Colorado Boulder, Boulder, Colorado, USA; 2 Howard Hughes Medical Institute, Chevy Chase, Maryland, USA

**Keywords:** PARN, USB1, TOE1, non-coding RNA regulation, deadenylases

## Abstract

The levels of non-coding RNAs (ncRNAs) are regulated by transcription, RNA processing, and RNA degradation pathways. One mechanism for the degradation of ncRNAs involves the addition of oligo(A) tails by non-canonical poly(A) polymerases, which then recruit processive sequence-independent 3′ to 5′ exonucleases for RNA degradation. This pathway of decay is also regulated by three 3′ to 5′ exoribonucleases, USB1, PARN, and TOE1, which remove oligo(A) tails and thereby can protect ncRNAs from decay in a manner analogous to the deubiquitination of proteins. Loss-of-function mutations in these genes lead to premature degradation of some ncRNAs and lead to specific human diseases such as Poikiloderma with Neutropenia (PN) for *USB1*, Dyskeratosis Congenita (DC) for *PARN* and Pontocerebellar Hypoplasia type 7 (PCH7) for *TOE1*. Herein, we review the biochemical properties of USB1, PARN, and TOE1, how they modulate ncRNA levels, and their roles in human diseases.

Gene expression in eukaryotes is regulated at multiple levels. While transcription regulation plays an important role, the regulation of RNA processing and degradation are also important in modulating the levels of both mRNAs and non-coding RNAs (ncRNAs) (1).

After transcription, 3′-end modifications of mRNA molecules can modulate the fate of newly synthesized RNAs. The 3′ ends of mRNAs are dynamically changed by the opposing effects of poly(A) polymerases and exonucleases, which can affect all aspects of mRNA metabolism (2). For example, the addition of poly(A) tails to mRNAs promotes their processing, export, and translation (3, 4, 5, 6, 7). Moreover, 3′ poly(A) tails on mRNAs can increase their stability by reducing the rate of decapping and/or inhibiting access to 3′ to 5′ exonucleases (8).

Similarly, 3′-end modifications of ncRNAs can regulate RNA processing and/or degradation. For instance, the addition of CCA to tRNA and uridylation of U6 small nuclear RNA (snRNA) are involved in RNA maturation (9, 10, 11), and the monouridylation of let-7 pre-miRNA by TUT4/TUT7 enhances its processing into mature let-7 (12). In contrast, other polymerases can promote degradation. For example, non-canonical poly(A) polymerases of the TRAMP complex interact with the nuclear exosome complex and are involved in the 3′-end processing and degradation of rRNAs and snoRNAs (13, 14, 15). Likewise, the uridylation of target RNAs, such as uridylation of let-7 pre-miRNA by TUT4/TUT7, or histone mRNAs (16), can recruit exonucleases for RNA degradation (13, 14, 15, 17, 18, 19, 20, 21).

Oligo(A) tail addition can also promote the degradation of some ncRNAs. For example, oligoadenylation by PAPD5/PAPD7 of miRNAs, human telomerase RNA (hTR), Y RNAs, ribosomal RNAs (rRNAs), small nucleolar RNAs (snoRNAs), small Cajal body-specific RNAs (scaRNAs), and small nuclear RNAs (snRNAs) can recruit exonucleases to degrade the RNAs (22, 23, 24, 25, 26, 27, 28, 29, 30). Similarly, PAPD5-mediated adenylation has been proposed to destabilize miR-21 and hTR RNAs in human cancer cell lines (22, 25, 26, 27, 28).

As oligo(A) tails can promote RNA degradation, it is not surprising that a set of deadenylases can remove oligo(A) tails and thereby stabilize some ncRNAs. To date, there are three such ncRNA deadenylases that can regulate ncRNAs in this manner including the poly(A) specific ribonuclease (PARN), USB1 (also called Mpn1), and Target of Erg1 (TOE1, also called Caf1z). Recent studies have shown that these enzymes regulate the stability of several ncRNAs in mammalian cells, such as hTR, Y RNAs, piwi-interacting RNAs (piRNAs), and miRNAs by removing poly(A) tails added by PAPD5/7. The poly(A) tail removal limits the recruitment of exonucleases DIS3L, DIS3L2, and/or the nuclease exosome (22, 23, 24, 25, 26, 27, 28, 29, 30, 31, 32, 33, 34, 35, 36, 37).

The process of oligo(A) tail addition and removal regulating the degradation of ncRNAs can be considered analogous to the control of protein degradation by ubiquination and deubiquination (Fig. 1). In this analogy, the oligo(A) polymerases such as PAPD5/7 are analogous to E3 ubiquitin ligases. Moreover, the recent results showing that PARN, TOE1, and USB1 stabilize different ncRNAs by removing these oligo(A) tails suggest this group of enzymes functions analogously to deubiquitinases. This suggests an emerging and unappreciated commonality of function for the USB1, TOE1, and PARN enzymes.

**Figure 1:**
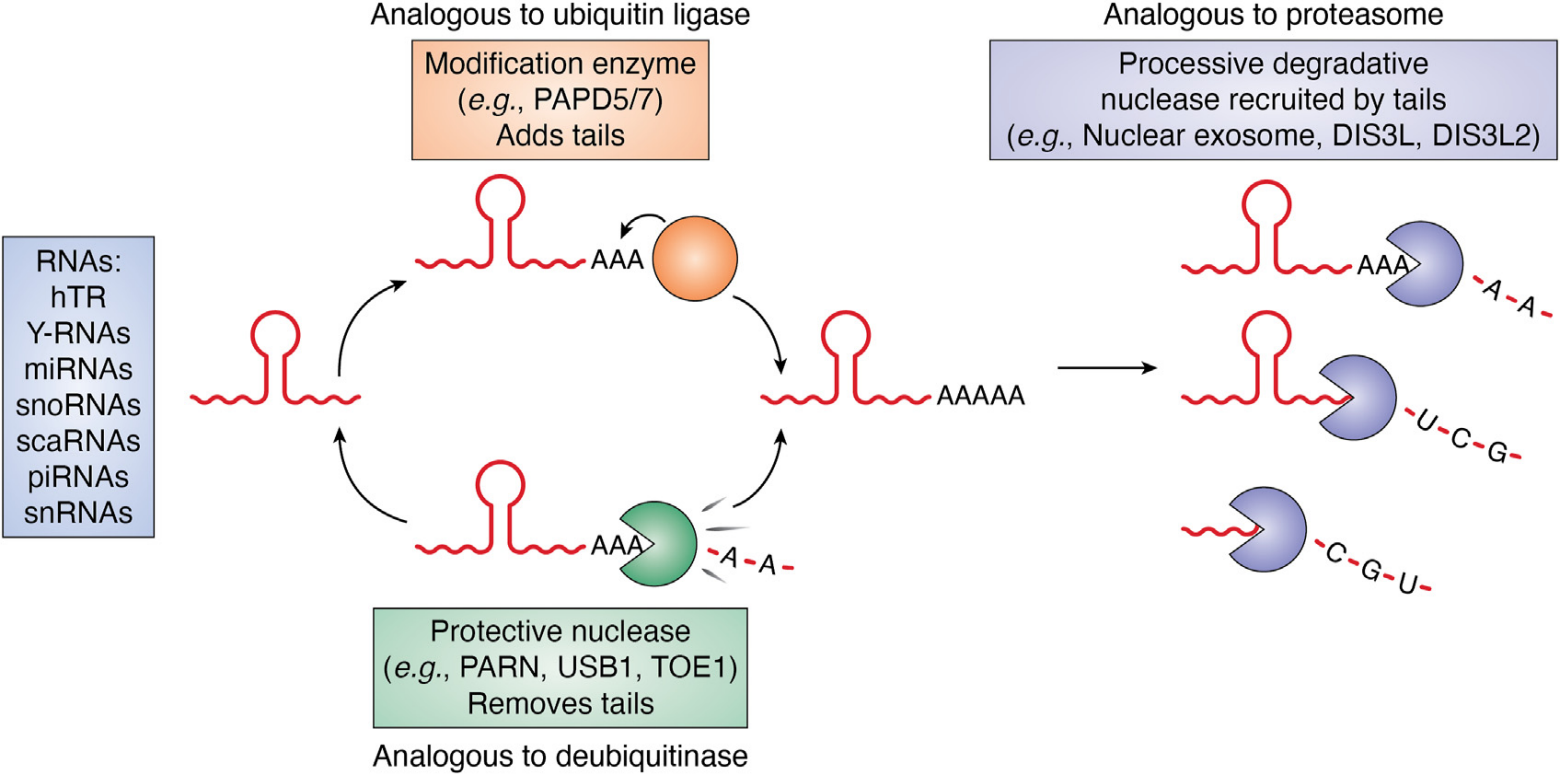
**Analogy of adenylation and deadenylation of ncRNAs to ubiquination and deubiquination.** The process of regulating ncRNA degradation by addition and removal of the poly(A) tail can be considered analogous to the control of protein degradation by ubiquination and deubiquination.

Consistent with a commonality of function, USB1, PARN, and TOE1 are each connected to a specific human disease (32, 38, 39, 40, 41, 42, 43, 44, 45, 46, 47, 48). For example, loss-of-function mutations in *USB1* (USB1 mutants) lead to the genetic disorder Poikiloderma with Neutropenia (PN), an autosomal-recessive bone marrow failure (BMF) syndrome with marked clinical overlap with Dyskeratosis Congenita (DC) (49). In contrast, loss-of-function mutations in *PARN* (PARN mutants) cause a severe form of DC called Hoyeraal-Hreidarsson syndrome, which causes abnormally short telomeres and congenital defects (38, 39, 40, 41) and idiopathic pulmonary fibrosis (IPF) (40). Finally, biallelic loss-of-function mutations in *TOE1* (TOE1 mutants) cause Pontocerebellar Hypoplasia type 7 (PCH7), a unique recessive syndrome characterized by neurodegeneration with ambiguous genitalia (32, 45).

Herein, we review the properties of USB1, PARN, and TOE1, and their roles in RNA regulation. As mutations in these enzymes lead to human diseases, understanding their functions and targets may provide insights into treatments for these diseases.

## The USB1, PARN, and TOE1 enzymes

**Figure 2:**
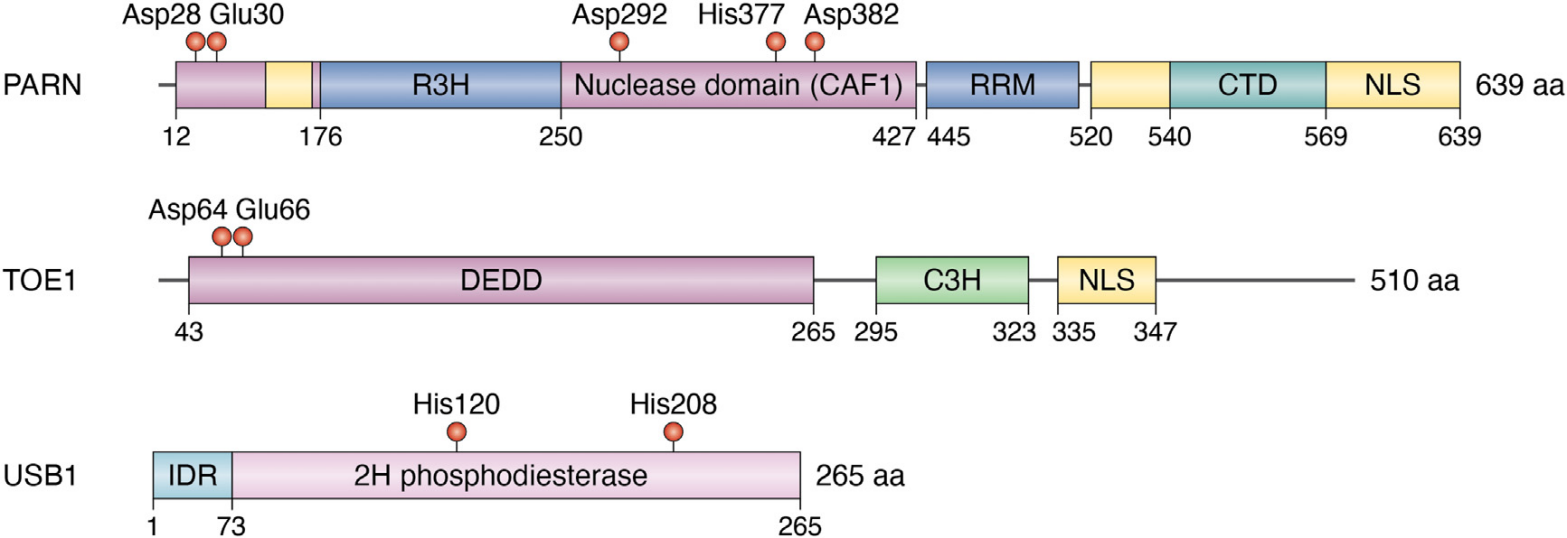
**Cartoons illustrating the domains of USB1, PARN, and TOE1**.

USB1 is a member of the 2H phosphodiesterase superfamily, which can be subdivided into H x T and H x S enzymes, the latter of which contains USB1 (50). The 2H phosphodiesterase superfamily is an enzyme family with 2′,3′-cyclic or 1′,2′-cyclic phosphodiesterase (CPDase) activity, 3′-5′ or 2′-5′ phosphodiesterase activity, or 2′,5′-RNA ligase activity. The active sites of these enzymes all utilize two catalytic histidines within the central H x S/T tetrapeptide motifs that act as a general acid and base, while the serine or threonine residues help coordinate substrates and assist in transition state stabilization (51, 52, 53, 54, 55, 56, 57). In the USB1 family members, these key histidines are His120 and His208 in human USB1 (PDB ID 6D30 and 6D2Z) (Fig. 2), His109 and His199 in fission yeast (*S. pombe*) USB1, and His133 and His231 in budding yeast (*Saccharomyces cerevisiae*) (PDB ID 5UQJ). Although the overall sequence conservation can be rather low between family members, all known crystal structures of 2H family members, including USB1, display a characteristic fold with conserved terminal and transit lobes and the H x S/T motifs centrally positioned in a substrate binding cleft (53, 57, 58, 59, 60, 61, 62, 63, 64, 65, 66).

In contrast, PARN and TOE1 belong to the DEDD protein superfamily, one of the six exoribonuclease superfamilies (67, 68). Exoribonucleases in the DEDD superfamily are part of the much larger exonuclease superfamily, containing the proofreading domains of many DNA polymerases as well as other DNA exonucleases (69, 70). DEDD-type nucleases are named after their conserved catalytic Asp and Glu residues and include several other conserved residues distributed in three separate sequence motifs (70). The nucleases of this superfamily coordinate two metal ions essential to their catalytic mechanism (71). The DEDD superfamily can be divided into two subgroups, DEDDy and DEDDh, which are distinguished by whether the fifth conserved residue is a tyrosine or a histidine (67). Based on its crystal structure, PARN was determined to belong to the DEDDh subfamily (67).

PARN consists of three domains: an RNA recognition motif domain (RRM), a nuclease domain, and an R3H domain (Fig. 2). Mutational analyses showed that four conserved DEDD residues in PARN (Asp28, Glu30, Asp292, and Asp382) are essential for the catalytic activity of PARN and are required for the binding of divalent metal ions to PARN (72). Moreover, when substituting His377 by Ala in C-terminal truncated PARN, PARN’s activity is inhibited, suggesting that His377, as the fifth conserved residue, is also essential for the catalytic activity of PARN (73).

There are modest studies on TOE1 biochemical properties compared to PARN. Initial inspection of the human genome revealed TOE1 as a distant homolog of yeast Caf1p, which encodes an mRNA deadenylase subunit (74, 75). However, through evolutionary distance analysis, TOE1 shows greater similarity to PARN than to Caf1 (76), which led to TOE1 being classified as a member of the DEDDh family. The TOE1 protein consists of a unique C3H-type zinc finger domain, a DEDD nuclease domain, and an arginine-rich predicted basic nuclear localization signal (NLS) (Fig. 2) (77). Point mutations Asp64Ala and Glu66Ala in the nuclease domain of TOE1 showed little deadenylase activity *in vitro*, demonstrating these residues are important for TOE1’s enzymatic activity (77).

## Biochemical roles of USB1, PARN, and TOE1 in RNA regulation

### Biochemical roles of USB1

USB1 was first shown to affect U6 snRNA maturation (57). U6 snRNA is a part of the spliceosome, a large and highly dynamic complex that acts to remove introns from precursor mRNAs (43, 51, 78, 79). Nascent human U6 and U6atac snRNA transcripts are transcribed with a heterogeneous polyuridine 3′ end, owing to the stochastic nature of RNA polymerase III terminations (80, 81). While TUT1 catalyzes 3′ polyuridylation of U6 and U6atac snRNAs, USB1 removes 3′ uridines from U6 (57). More specifically, *in vitro* experiments showed that USB1 can remove uridine nucleotides from the 3′ end of U6 snRNA and catalyzes a formation of terminal 2′, 3′ cyclic phosphate, which stimulates the binding of Lsm2-8 and leads to the formation of U6 snRNPs (58, 64, 82, 83, 84).

Human and budding yeast USB1 appear to process U6 snRNAs differently, despite sharing highly similar structures (57, 58, 64, 85). *In vitro* analyses showed that the human USB1 post-transcriptionally removes uridine and adenosine nucleosides from the 3′ ends of spliceosomal U6 snRNAs, and can catalyze terminal 2′, 3′-cyclic phosphate formation (57, 58, 64, 85). USB1 measures the appropriate length of the U6 oligo(U) tail by reading the position of a key adenine nucleotide (A102) and pausing five uridine residues downstream (51, 57, 58, 64, 86). In *S. cerevisiae*, an unbiased genetic screen revealed that the yeast ortholog of *USB1* is essential for U6 snRNA biogenesis and cell viability (78). In *S. cerevisiae*, USB1 mainly removes a single nucleotide from U6, leaving a 3′ monophosphate which strongly inhibits further processing (64). This argues that *S. cerevisiae* USB1 has 2′-CPDase activity that hydrolyzes the 2′,3′-cyclic phosphate product into a 3′ monophosphate, which is not exhibited in human USB1.

Crystallography studies showed that even with low similarity in sequence identity (<20%), humans and *S. cerevisiae* USB1 share highly similar structures (PDB ID 6D31, 4H7W, and 5UQJ) (57, 64). Moreover, these structures of human USB1 bound to nucleotides and intact RNA, along with kinetic analyses, and QM/MM simulations, which is a type of molecular stimulation method (87), have provided insights into its catalytic mechanism (57, 64). However, despite their similar structure, the mechanism of *S. cerevisiae* USB1’s 2′-CPDase activity remained poorly understood. By comparing *S. cerevisiae* USB1 (PDB ID 5UQJ) and *K. marxianus* USB1 structures (PDB ID 6PFQ), it has been suggested that the CPDase activity comes from a loop structure that is conserved in yeast and forms a distinct penultimate (n-1) nucleotide binding site (85). This suggested that the CPDase activity in yeast USB1 is related to the loop architecture that many yeast species possess but is absent from the human USB1.

**Figure 3:**
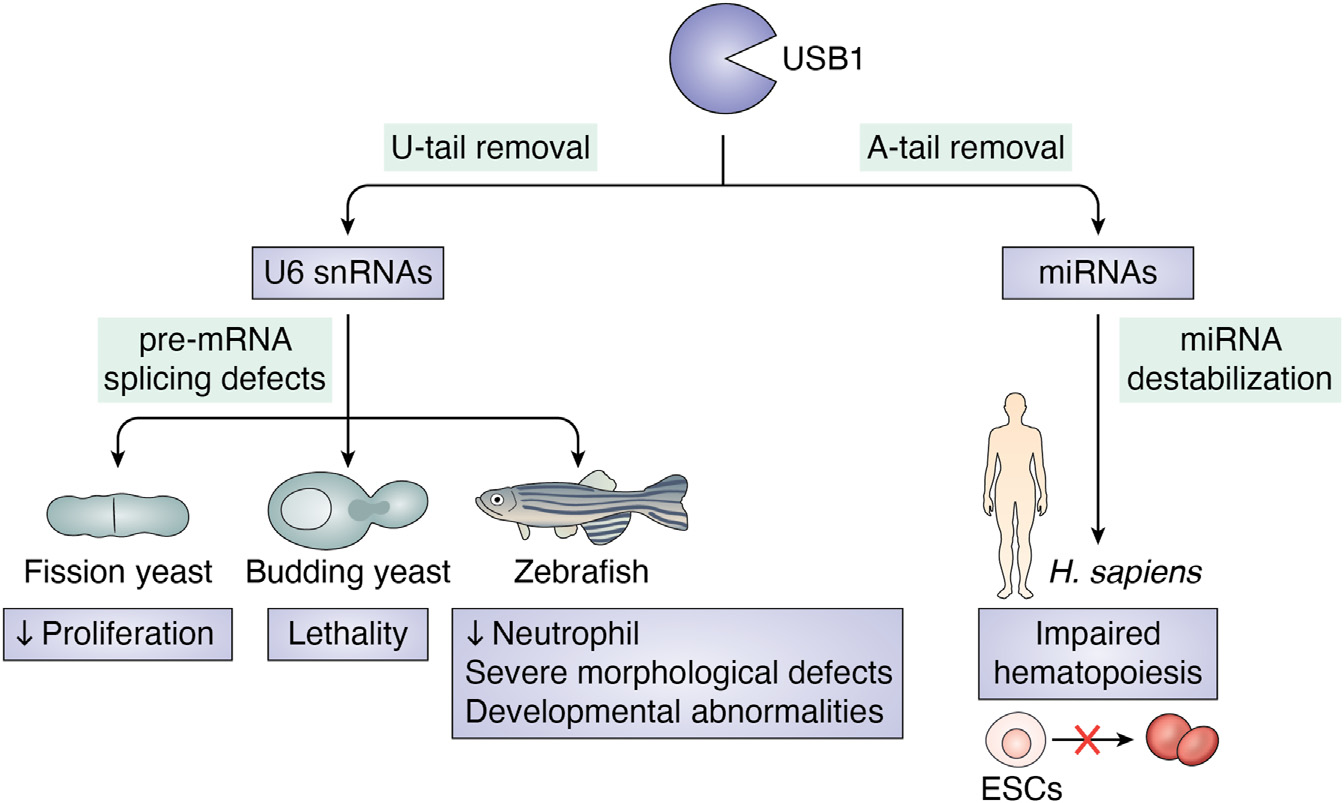
**Substrates of USB1 and USB1-related phenotypes.** USB1 removes poly(U) tails from U6 snRNAs and poly(A) tails from miRNAs (31, 57, 58, 64, 78, 85)**.** USB1-deficiency leads to pre-mRNA splicing defects and miRNA destabilization, resulting in developmental defects of various species and hematopoiesis (31, 42, 43, 51, 58, 78, 88).

Despite the related role for USB1 in U6 and U6atac snRNA processing, USB1 defects show distinct phenotypes in different species (Fig. 3). In budding yeast, *USB1* deletion leads to cell death (58, 78), while in the fission yeast strand, the Δ*USB1* cells only showed reduced proliferation (51). In both yeast species, cells with *USB1* depletions (where *USB1* is expressed from a glucose-repressible galactose promoter) show global defects in pre-mRNA splicing, and this can be reversed by over-expressing U6 (51, 58, 78). This suggests that these yeast phenotypes are probably linked to U6 processing by USB1, leading to a subsequent defect in proper pre-mRNA splicing.

In more complex organisms, USB1 can affect other processes including hematopoiesis (Fig. 3) (31, 87, 88). This is relevant for USB1 since people with loss-of-function alleles of *USB1* have PN syndrome, which includes defects in the production of neutrophils (88). In PN-modeled zebrafish, morpholino *USB1* suppression causes pigmentation and osteochondral defects, severe morphological defects, including a bent tail, thin yolk extension, reduced body length, and a significantly decreased number of neutrophils (87, 88). Thus, USB1-deficient embryos display developmental abnormalities recapitulating some of the properties of the human syndrome (88). In this zebrafish model, *USB1* KD also causes splicing defects and a decrease in the formation of aberrant transcripts (87, 88). Specifically, the splicing of genes involved in neutrophil differentiation and development was aberrant in the morphant. Real-time PCR analyses of stage-specific markers showed defects of primitive hematopoiesis (87, 88). Together, these studies demonstrate the intrinsic requirement of USB1 for hematopoiesis and raise the possibility this could be affected by changes in pre-mRNA splicing.

In humans, USB1 also regulates hematopoietic development, but this appears to be primarily through deadenylating miRNAs instead of directly affecting splicing (Figs. 3 and 6*A*) (31, 58). The possibility that USB1 affects miRNAs comes from several observations in experiments done with a pathogenic *USB1* mutation introduced into hESCs (31). First, while most ncRNAs, mRNAs, and U6 snRNA levels were not affected by the *USB1* mutation, some key miRNAs affecting hematopoietic development were decreased. Second, when these miRNAs were over-expressed, the USB1-dependent defect in hematopoiesis was rescued. Third, there was an increase in short A tails on miRNAs in the USB1 mutant, and those tails were reduced by inhibition of the PAPD5/7 adenylase. Finally, inhibition of PAPD5/7 adenylase rescued miRNA levels and the defect in development seen in the USB1 mutant cells. While USB1 generally acts on U-tails, it can remove poly(A) tails *in vitro* (31, 57). These results suggest that one function of USB1 is to remove oligo(A) tails of miRNAs and thereby regulate human hematopoiesis.

USB1 may regulate hematopoiesis in a tissue-specific manner. USB1 is required for U6 and U6atac snRNA processing and there are observed defects in pre-mRNA splicing in PN-modeled zebrafish (51, 78, 87, 88). However, in PN-modeled zebrafish, there was no difference in U6 snRNA levels between wild-type and USB1-deficient embryos and only the genes involved in neutrophil differentiation and development showed splicing defects, not the hematopoietic precursors and erythroid-specific genes (87). These observations are consistent with the data from human PN that lymphoblastoid cells do not exhibit reduced U6 snRNA levels and have normal pre-mRNA splicing (58) and USB1 acts as a miRNA deadenylase to regulate hematopoiesis (31). These data suggested that USB1 might affect both splicing by U6 processing and miRNA stability by removing 3′-end adenylated tails added by PAPD5 to regulate hematopoiesis in a tissue-specific manner, which is supported by high levels of *USB1* expression in hemopoietic tissue (31).

### Biochemical roles of PARN

PARN is a poly(A) specific nuclease, first identified in extracts of *Xenopus* oocytes (89). PARN removes poly(A) tails from RNAs and releases 5′ AMP as a reaction product. It is suggested that the homodimer of PARN functions as a structural unit for its enzymatic activity since the substitution of Ile113, Phe123, or Phe127, important residues for homodimerization, inactivated or reduced PARN’s activity significantly (73). PARN has been demonstrated to preferentially cleave a poly(A) substrate with a free 3′-OH group (89, 90). This conclusion was supported by crystal structure showing that Glu30 specifically interacts with the 3′ hydroxyl group of the ribose (PDB ID 2A1R) (73). This observation suggested that Glu30 may act both as the catalytic residue and also confer specificity for the recognition of the 3′ poly(A) tail.

Among all deadenylases, PARN is unique as it can bind both the cap structure and the poly(A) tail during deadenylation (91, 92, 93, 94, 95). PARN recognizes and binds m7GpppG through Trp residue in the RRM domain (Trp475 in human and Trp468 in mouse) while the R3H domain helps to stabilize PARN (95, 96, 97). PARN’s activity was higher when processing RNA with 5′-cap structure compared to noncapped RNA substrates and the addition of free m7GpppG cap analog inhibited poly(A) degradation *in vitro*, suggesting that 3′-end poly(A) removal is linked to 5′ end cap structure of the RNA substrates (90, 91, 93). One unclear functional property of PARN is its specificity for poly(A). Biochemical studies have shown that PARN degrades poly(A) most efficiently and poly(U) under certain conditions but with essentially no activity on poly(C) or poly(G) (89, 90). How PARN achieves specificity for poly(A) is unclear as there are no H-bonding interactions between adenine bases and the protein (PDB ID 2A1R) (73). Interestingly, the DEDD deadenylase enzyme Pan2 in complex with RNA also does not exhibit any canonical base-specific contacts (PDB ID 6R9O), and instead achieves poly(A) specificity through intrinsic stacked, helical confirmation of poly(A) RNA (98). This suggests that instead of the canonical mechanisms of sequence-specific RNA recognition, PARN may utilize a structure-based mechanism similar to Pan2 to achieve specificity for poly(A) tails.

**Figure 4:**
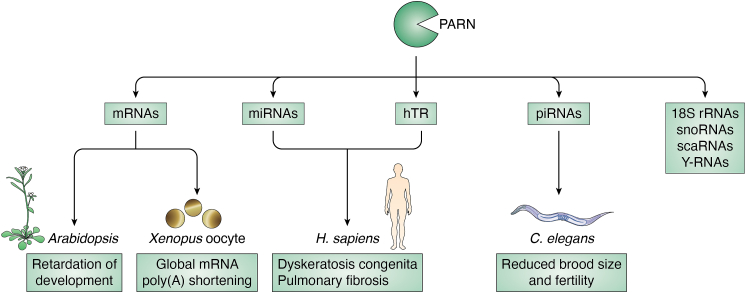
**Substrates of PARN and PARN-related phenotypes.** PARN removes poly(A) tails from mRNAs, miRNAs, hTR, piRNAs, 18S rRNAs, snoRNAs, scaRNAs, and Y RNAs (22, 23, 24, 25, 26, 27, 29, 30, 37, 106, 107, 114). PARN mutants show dysregulation of these RNAs and lead to developmental defects in higher plants, zebrafish, *C.elegans*, and DC and pulmonary fibrosis in humans (37, 38, 39, 40, 41, 46, 47, 89).

Recent studies have identified several targets of PARN. Although PARN was suspected as a key regulator for mRNAs due to its preference for m7G-cap and its role in global poly(A) shortening during *Xenopus* oocyte (89, 99, 100), PARN was shown to predominantly localize to the nucleolus and cytoplasmic foci and process ncRNAs in HeLa cells, such as 18S rRNAs, snoRNAs, hTR, scaRNAs, piRNAs, Y RNAs, and miRNAs (Fig. 4) (22, 23, 24, 25, 26, 27, 29, 30, 98, 101, 102, 103, 104, 105, 106).

PARN also plays important role in development (Fig. 4). PARN is essential in plant development (107, 108, 109). Specifically, PARN mutant embryos showed developmental defects in higher plants, which has been proposed to be due to a failure to remove the 3′ poly(A) tails on specific subsets of mRNAs (110). In *C. elegans*, PARN mutants show reduced brood size and fertility compared to wild-type animals and accumulated untrimmed piRNAs with 3′ extensions (37, 76). Moreover, during *Xenopus* oocyte maturation, PARN was shown to cause global poly(A) shortening (89, 99, 100). These studies indicated that PARN deadenylase activity is crucial for the development of various species.

PARN deadenylase activity is involved in cancers and human diseases. A recent study found that in acute lymphocytic leukemia and acute myeloid leukemia, PARN’s expression levels were increased compared to non-malignant clinical samples (111, 112). In lung cancer, expression of PARN is associated with increased overall survival (110). Moreover, mutations in *PARN* are associated with DC and the familial form of idiopathic pulmonary fibrosis (IPF), an age-related disease featuring processive lung scarring (38, 39, 40, 41, 47). Both of these diseases are characterized by defects in telomerase activity and shorter telomeres (38, 39, 40, 41). Since PARN acts as a deadenylase to remove 3′ oligoadenylated ends of hTR, this suggests a link between PARN, hTR deadenylation, and the causes of DC and IPF (Figs. 4 and 6*B*) (22, 29, 111).

PARN requires binding partners for proper regulation in cells. The binding partners can either repress or activate the enzyme’s activities. For example, the interaction of the poly(A) tail with poly(A) binding proteins (PABPs) and/or the interaction of cap-binding proteins such as eIF4E and CBP80 with the 5′ cap structure has been shown to repress PARN and protect RNA substrates (113, 114). On the other hand, several RNA-binding proteins help to recruit PARN to the target RNAs for poly(A) removal. For instance, the CUG-binding protein (CUG-BP) can bind to cFos and TNFα mRNA and stimulate the poly(A) tail shortening of these RNAs by recruiting PARN (115). Similarly, miR-125b-loaded miRISC has been suggested to contribute to the specific recruitment of PARN to TP53 mRNA to regulate p53 levels (116). Finally, human tristetraproline (TTP) activates PARN activity when transfected in HEK293 extracts and this activation requires the binding of TTP to RNAs (117).

### Biochemical roles of TOE1

TOE1 is a deadenylase that was first identified as a target of Erg1, an immediate early transcription factor. One function of Erg1 is to decrease the growth and tumorigenic potential of several tumor cell types (118, 119) and TOE1 was shown to be accountable for the growth inhibitory effect of Erg1 (74). Since TOE1 shows high similarity to PARN through evolutionary distance analysis (76), one could hypothesize that TOE1 may act redundantly with PARN in removing 3′ end tails of RNAs. However, in HeLa cells, TOE1 was shown to localize to Cajal bodies, while PARN is primarily in the nucleoli, indicating that TOE1 and PARN have distinct subcellular locations (29, 30, 98).

TOE1 exhibits deadenylation on RNA substrates and shows a preference for poly(A) (77). However, TOE1 catalyzes rapid deadenylation followed by a slower 3′-to-5′ exonucleolytic decay of non-poly(A) sequences, which is distinct from PARN (77).

**Figure 5:**
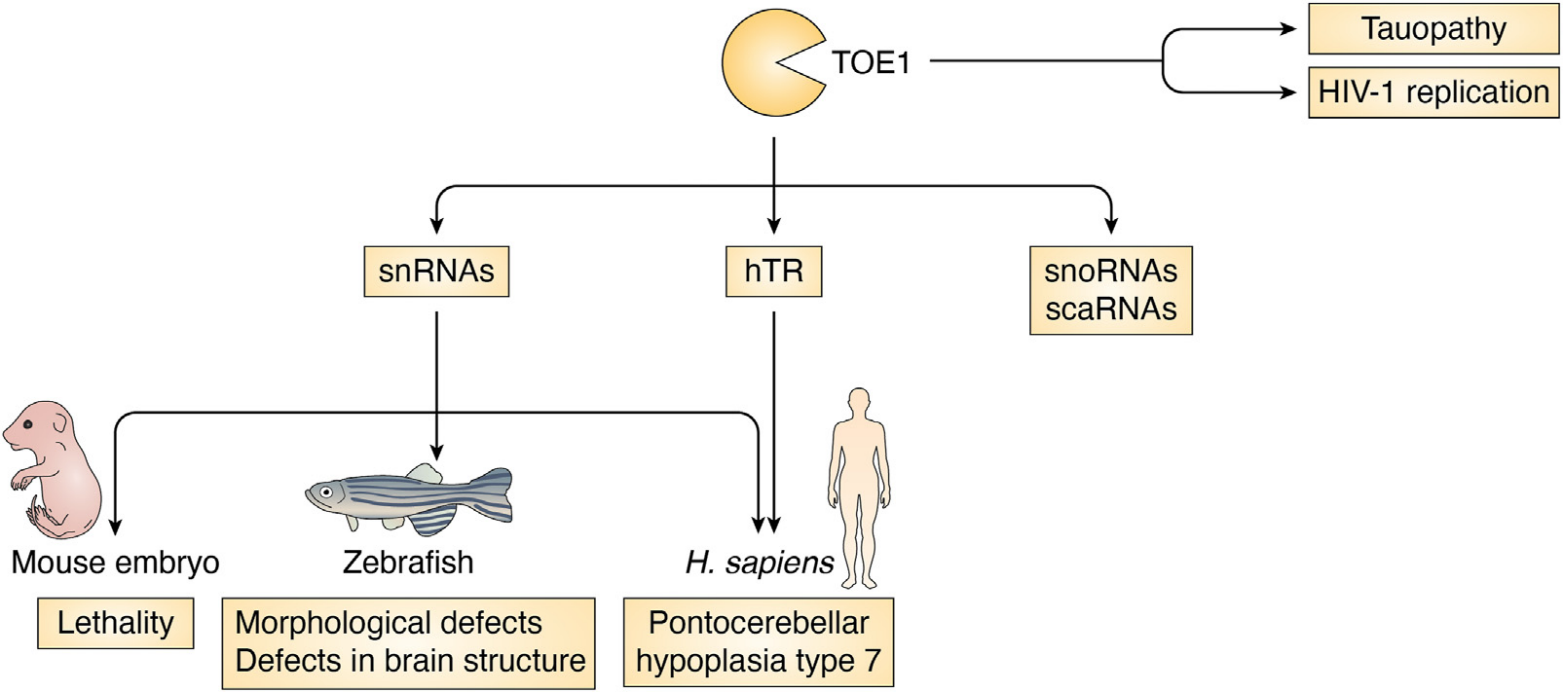
**Substrates of TOE1 and TOE1-related phenotypes.** TOE1 removes poly(A) tails from snRNAs, hTR, snoRNAs, and scaRNAs (30, 32, 34). TOE1 mutants in PCH7 patients and PCH7-modeled mice and zebrafish lead to extended adenylated 3′ ends of hTR and snRNAs, resulting in developmental defects and defects in brain structures (32, 45).

TOE1 has been shown to deadenylate several ncRNAs, including snRNAs (32). More specifically, TOE1 promotes the maturation of all regular RNA polymerase II transcribed snRNAs of the major and minor spliceosomes by removing 3′ oligo(A) tails and preventing nuclear exosome targeting (33). However, TOE1 removal of oligo(A) tails on snRNAs and snoRNAs can provide a mechanism for RNA quality control (33). TOE1 does show some overlap of substrates with PARN, and together both these enzymes can act on snoRNAs, scaRNAs, and hTR (Fig. 5) (30, 34)).

TOE1 expression also shows effects on development. Over-expression of TOE1 negatively affects the growth of HEK293 and H4 cells by altering the cell cycle through the induction of p21 (34, 74). On the contrary, TOE1 KD causes developmental arrest during the morula-to-blastocyst transition in mice by upregulating p21 (120). These studies suggest that TOE1 regulating p21 may be tissue-specific and further investigation of the molecular mechanism of TOE1 is needed for a better understanding of TOE1’s role in development.

**Figure 6:**
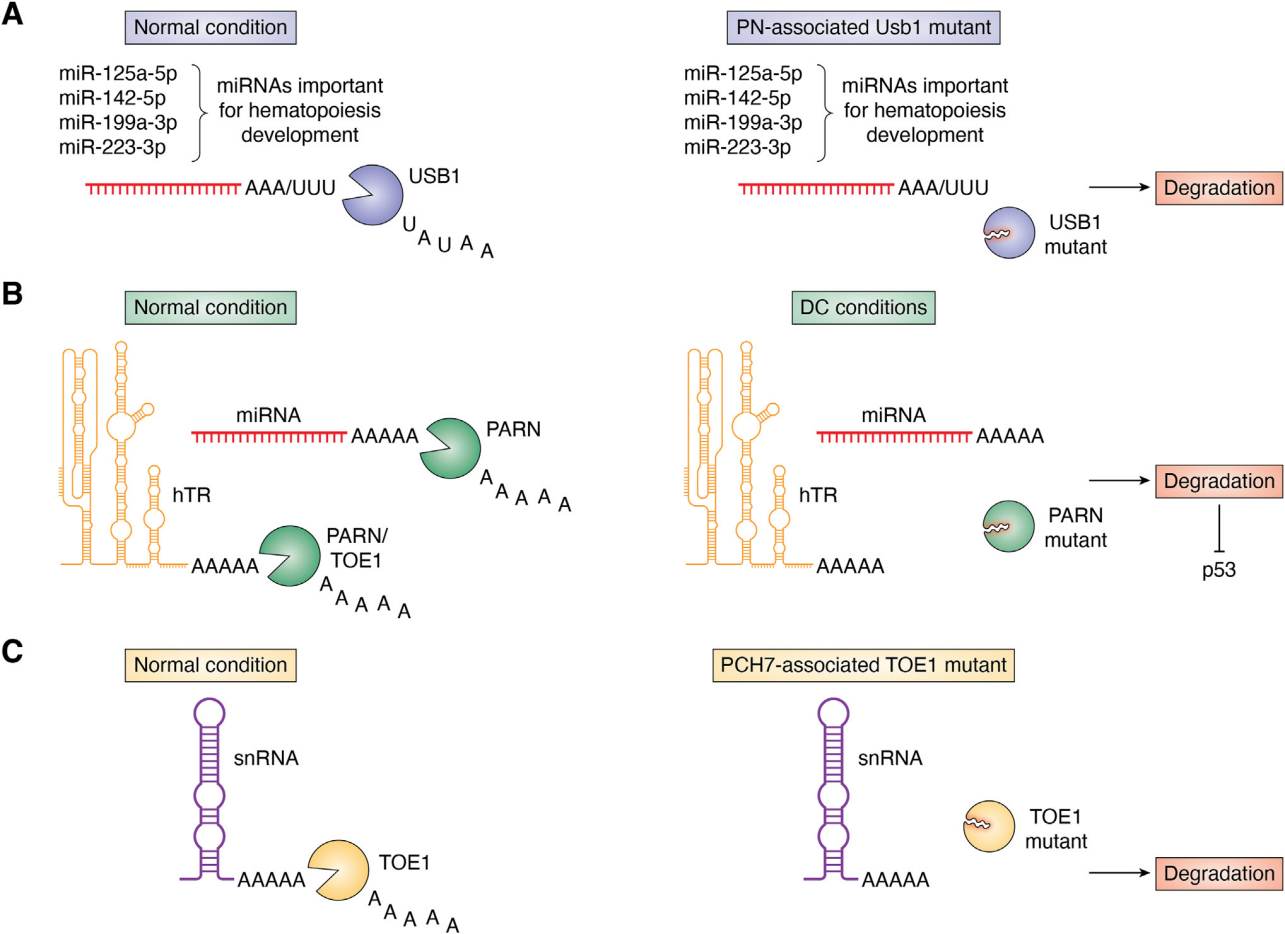
**Model depicting the regulation of non-coding RNA stability through ribonucleases in different diseases.***A*, miRNAs that are important for hematopoiesis development are stabilized by USB1 in normal conditions while are targeted for degradation in Poikiloderma with Neutropenia disease where *USB1* is mutated (31, 42, 78). *B*, hTR and miRNAs are stabilized by PARN and/or TOE1 in normal conditions while are targeted for degradation in Dyskeratosis Congenita disease where *PARN* is mutated (22, 24, 34, 38, 41, 46). *C*, snRNAs are stabilized by TOE1 in normal condition while are targeted for degradation in Pontocerebellar Hypoplasia type 7 where *TOE1* is mutated (32, 45).

TOE1 is involved in PCH7 (Figs. 5 and 6*C*). This was first suggested by the identification of various *TOE1* mutations in PCH7 patients (32, 45). Moreover, mouse embryos with homozygous *TOE1* frameshift mutations showed uniform lethality demonstrating *TOE1* is required for mouse development. Furthermore, in a PCH7-disease model using zebrafish, knockdown of the single *TOE1* orthologue led to reproducible microcephaly, small eye, and curly tail phenotype in 90% of embryos with defects in the brain structure of the midbrain, cerebellum, and hindbrain by 48 h post-fertilization. This phenotype was rescued by co-injection of human *TOE1* mRNA but not the catalytically inactive DE mutant- or patient mutation-encoding mRNAs, suggesting that reduced expression of TOE1 leads to neurodegeneration and PCH-like brain defects *in vivo* (32). Moreover, TOE1 DE-associated snRNAs are predominantly enriched for untemplated 3′ adenosine (32), suggesting a link between PCH7 and TOE1 deadenylase activity on the processing of snRNA 3′ ends.

TOE1 is also suggested to modulate HIV-1 infection (Fig. 5). TOE1 was shown to directly interact with the HIV viral transactivation response element as part of an inhibitory mechanism (121). Moreover, TOE1 can be secreted following immune response activation and exhibits the ability to spontaneously cross the plasma membrane and penetrate cells in culture (121). Interestingly, exogenously added TOE1 can also inhibit HIV-1 LTR (long terminal repeat) transactivation, retaining HIV-1 inhibitory activity (121). Both TOE1’s antiviral potency and its cell-penetrating capability have been identified to lie within the 35-amino-acid region containing the nuclear localization signal (NLS) (121). This suggests that TOE1 may have other functions besides ncRNA deadenylation.

TOE1 was also shown to be involved in tauopathy. In *C. elegans*, loss of *parn-2*, an ortholog of *TOE1*, partially suppressed tauopathy (122). Moreover, this might be relevant to human disease since Alzheimer’s disease patients with low TOE1 levels exhibit significantly increased pathological tau deposits (122). Interestingly, blocking 3′ end poly(A) extensions with cordycepin exacerbated tauopathy in human cultured cells (122). Together, these data suggest that there is a link between tauopathy, TOE1, and poly(A) RNA metabolism.

Similar to PARN, TOE1 has been shown to associate with several proteins, including hCcr4d, DKC1 (TERC subunit), and spliceosomal proteins (32, 34, 77); however, whether these interactions modulate TOE1 function has not been determined.

### Human diseases are caused by failure to deadenylate ncRNAs

A striking hallmark is that loss-of-function mutations in *USB1*, *PARN*, and *TOE1* are all involved in human diseases featuring abnormal 3′ end extensions of ncRNAs (32, 38, 39, 40, 41, 42, 44, 45, 46, 47, 48). Thus, an understanding of these disease mechanisms may suggest possible manipulations to restore ncRNAs for therapeutic improvements.

Loss-of-functions mutations in *C16orf57*, which encodes USB1, in humans leads to PN, which is a rare, autosomal recessive skin condition (OMIM 604173). To date, there are 38 PN patients reported with 19 different mutations in *C16orf57* (43, 49, 123). Based on the bioinformatic prediction, all *C16orf57* mutations impair the protein structure by either removing one or both tetrapeptide motifs, or destroying the symmetry of the native folding. Mutations in *C16orf57* gene also produce phenotypes with marked clinical overlap with DC and Rothmund-Thomson syndrome (RTS), a poikiloderma that is sometimes confused with PN (49). However, unlike patients with DC, which is often caused by mutations in factors involving telomere maintenance, telomeres in patients with PN are not significantly shortened, and therefore telomere length represents a clear distinguishable feature for the correct diagnosis of these different BMF syndromes (48). RTS can also be distinguished from PN since RTS patients harbor mutations in the RECQL4 DNA helicase, which is involved in DNA repair and replication (124, 146). Thus, mutations in *C16orf57* can be used as a molecular marker for the precise diagnosis of PN.

In humans, it appears USB1 acts as a miRNA deadenylase to regulate hematopoietic development (Fig. 6*A*). A previous study showed that lymphoblastoid cells from PN patients do not exhibit reduced U6 snRNA levels and have normal pre-mRNA splicing (58). Recently, the analysis of human embryonic stem cells harboring PN-associated mutation c.531_delA in *C16orf57* showed that this mutation severely impairs human hematopoietic development (31). In this model system, it was demonstrated that hematopoietic failure in USB1 mutants is caused by dysregulated miRNA levels in hematopoiesis, due to a failure to remove destabilizing 3′ end oligo(A) tails added by PAPD5/7. This phenotype can be reversed by inhibition of PAPD5/7 with a PAPD5/7 inhibitor, RG7834 (31). Despite the widely appreciated role of USB1 in U6 maturation, this study identified a new role for USB1 in miRNA regulation and in PN disease.

Loss-of-function mutations in *PARN* also lead to a type of BMF. Specifically, mutations in *PARN* were found in a severe form of DC known as Hoyeraal-Hreidarsson syndrome and IPF (38, 39, 40, 41, 46, 47). DC is an inherited, life-threatening bone marrow failure disorder, caused by genetic defects in components of the telomerase holoenzyme in human cells and leads to age-related bone marrow failure and cancer. Most mutations associated with DC are found in genes encoding components of the telomerase enzyme complex including hTR, the telomerase RNP component dyskerin (*DCK1*), and the catalytic subunit TERC (124, 125). Moreover, it was shown that loss of PARN leads to defective 3′ end maturation of hTR, suggesting the link between PARN, hTR maintenance, and the causes of DC and IPF (22, 25, 26, 27, 30, 41, 111).

PARN deficiency negatively affects the stability of hTR and miRNAs, which is likely to cause severe phenotypes in DC and IPF (Fig. 6*B*). Several studies have shown that PARN inhibition leads to a failure to remove the poly(A) tail of hTR, leading to the recruitment of nuclear 3′ to 5′ exonuclease EXOSC10, which leads to hTR degradation (22, 25, 26, 27, 29, 30, 41, 111). Moreover, it was shown that PARN deficiency also leads to the accumulation of longer oligo(A) tails and affects the stability of several miRNAs, which upregulate p53 expression (24). Since chronic upregulation of p53 signaling would negatively affect cell growth and development, this could explain the severe phenotype of PARN deficiency in DC patients (24).

Mutations in *TOE1* were found in PCH7 (32, 45). Pontocerebellar hypoplasias (PCH) are a group of autosomal recessive neurodegenerative disorders with prenatal onset, mainly affecting the growth and survival of neurons in the cerebellar cortex, the dentate, inferior olivary, and ventral pontine nuclei (126). To date, 10 subtypes of PCH have been identified (32, 126, 127, 128, 129, 130, 131, 132, 133, 134, 135, 136, 137, 138, 139, 140, 141). PCH7 is characterized by neurological deterioration, astrophy/hypoplasia of the pons and cerebellum, muscular hypotonia, and breathing abnormalities, combined with hypogonadism (142).

Patients with PCH7 harbor biallelic, loss-of-function mutations in *TOE1*, resulting in the accumulation of incompletely processed snRNAs (Fig. 6*C*). TOE1 is associated with pre-snRNAs and snRNAs associated with TOE1 catalytically inactive mutant, U1, U2, and U5 snRNAs contained longer tails than those associated with WT TOE1, suggesting that TOE1 plays a role as a 3′-5′ exonuclease for specific snRNA processing (32). There was an increase in the fraction of U1 and U2 snRNAs, and to a less extent, U5 snRNAs containing tails in patient-derived TOE1 defective fibroblast and NPC lines compared to unaffected relatives, suggesting that there might be a link between the cause of PCH7 and a key factor involved in incompletely processed snRNAs which are likely caused by loss-of-function *TOE1* mutations (32).

TOE1 and PARN may act non-redundantly on regulating and maintaining hTR biogenesis in diseases (Fig. 6*B*). It is interesting to note that cerebellum hypoplasia also manifests in patients with telomere-related diseases, such as Hoyeraal-Hreidarsson and Revesz syndromes (142, 143). It has been shown that TOE1 deficiency leads to the accumulation of hTR precursors, including oligoadenylated and 3′ end extended forms. However, TOE1 deficiency only affects telomerase activity and shortening, but not hTR levels (34). The current model speculated that most of the hTR poly(A) tails are removed by PARN in the nucleoli, then hTR transits to Cajal bodies for further processing by TOE1 and/or PARN, cooperative or sequentially (34). This explains why when TOE1 is deficient, there are more immature hTR precursors but no decrease in total hTR levels. Thus, understanding the precise mechanisms of how these deadenylases function may give insights into the development and pathogenesis of diseases such as DC and PCH7.

Inhibition of PAPD5/7 may be a potential therapy for PN and DC. Dysregulated miRNA and hTR levels in USB1 and PARN mutants contribute to hematopoietic failure, due to a failure to remove 3′ end adenylated tails added by PAPD5/7. Previous studies showed that modulation of miRNA 3′ end adenylation through genetic or chemical inhibition of PAPD5/7 rescues hematopoietic in USB1 mutants (31). Likewise, PAPD5 KD can rescue telomerase activity in PARN-deficient cells and restore defects in hematopoiesis (22, 144). Though there is no data of how PAPD5/7 affects snRNAs and PCH7 phenotypes, these studies suggest that inhibitors of PAPD5/7 might be a potential treatment for PN and DC, and possibly PCH7.

## Future outlooks

It is now appreciated that the balance of the addition and removal of oligo(A) tails can serve as a common pathway for regulating the stability of some ncRNAs. In this context, the poly(A) specific exonucleases USB1, PARN, and TOE1 have emerged as playing important roles in controlling the stability of ncRNAs. Looking forward there are outstanding issues to address. First, it will be important to understand why these enzymes are specifically required for some developmental transitions in both plants and mammals. Understanding these areas should shed new light on the roles of their biological roles and may identify new RNA substrates for these enzymes. Second, it will be important to determine whether therapeutic intervention in the ncRNA adenylation/deadenylation dynamic can be useful in therapeutic contexts. Currently, inhibition of adenylation by PAPD5/7 inhibitor has been suggested as possible therapy for DC and PN (31, 145). Alternatively, there may be other contexts where inhibition of ncRNA deadenylation might have therapeutic benefits (24), although testing these ideas will require the development of robust chemical inhibitors of PARN, USB1, and TOE1.

## Conflict of interest

The authors declared no conflict of interest for this review.
